# Genome-wide association study of SNP- and gene-based approaches to identify susceptibility candidates for lupus nephritis in the Han Chinese population

**DOI:** 10.3389/fimmu.2022.908851

**Published:** 2022-10-03

**Authors:** Kangkang Song, Xiaodong Zheng, Xiaomin Liu, Yujun Sheng, Lu Liu, Leilei Wen, Shunlai Shang, Yiyao Deng, Qing Ouyang, Xuefeng Sun, Qinggang Li, Pu Chen, Guangyan Cai, Mengyun Chen, Yuanjing Zhang, Bo Liang, Jianglin Zhang, Xuejun Zhang, Xiangmei Chen

**Affiliations:** ^1^ Department of Nephrology, The First Medical Centre, Chinese PLA General Hospital, Medical School of Chinese PLA, Chinese PLA Institute of Nephrology, State Key Laboratory of Kidney Diseases, National Clinical Research Center for Kidney Diseases, Beijing Key Laboratory of Kidney Diseases, Beijing, China; ^2^ Institute of Dermatology and Department of Dermatology at No.1 Hospital, Anhui Medical University, Hefei, China; ^3^ Department of Rheumatology, The First Medical Centre, Chinese PLA General Hospital, Beijing, China; ^4^ Institute of Dermatology and Department of Dermatology, Huashan Hospital, Fudan University, Shanghai, China; ^5^ Haihe Laboratory of Cell Ecosystem, Tianjin, China

**Keywords:** lupus nephritis, systemic lupus erythaematosus, genome-wide association study, susceptibility gene, gene-based analysis

## Abstract

**Background:**

Lupus nephritis (LN) is one of the most common and serious complications of systemic lupus erythaematosus (SLE). Genetic factors play important roles in the pathogenesis of LN and could be used to predict who might develop LN. The purpose of this study was to screen for susceptible candidates of LN across the whole genome in the Han Chinese population.

**Methods:**

592 LN patients and 453 SLE patients without renal damage were genotyped at 492,970 single nucleotide polymorphisms (SNPs) in the genome-wide association study (GWAS). Fifty-six SNPs were selected for replication in an independent cohort of 188 LN and 171 SLE without LN patients. Further quantitative real-time (qRT) PCR was carried out in 6 LN patients and 6 healthy controls. Gene-based analysis was conducted using the versatile gene-based test for GWAS. Subsequently, enrichment and pathway analyses were performed in the DAVID database.

**Results:**

The GWAS analysis and the following replication research identified 9 SNPs showing suggestive correlation with LN (*P*<10^-4^). The most significant SNP was rs12606116 (18p11.32), at *P*=8.72×10^−6^. The qRT-PCR results verified the mRNA levels of LINC00470 and ADCYAP1, the closest genes to rs12606116, were significantly lower in LN patients. From the gene-based analysis, 690 genes had suggestive evidence of association (*P*<0.05), including LINC00470. The enrichment analysis identified the involvement of transforming growth factor beta (TGF-β) signalings in the development of LN. Lower plasma level of TGF-β1 (*P*<0.05) in LN patients and lower expression of transforming growth factor beta receptor 2 in lupus mice kidney (*P*<0.05) futher indicate the involvement of TGF-β in LN.

**Conclusions:**

Our analyses identified several promising susceptibility candidates involved in LN, and further verification of these candidates was necessary.

## Introduction

Lupus nephritis (LN) is a major risk factor for morbidity and mortality in systemic lupus erythaematosus (SLE), which is an autoimmune disease characterized by the loss of self-tolerance and formation of nuclear autoantibodies and immune complexes (1, 2). Up to 60% of adult SLE patients will develops to LN and 10% to 30% of severe LN (Class III and above) cases might progress to end-stage renal disease (ESRD) (3). Indeed, patients with LN often have higher mortality and die earlier than the SLE patients without LN (4). LN is also associated with a 6-fold increase in mortality compared with the general population, and ESRD patients show a 26-fold increase (5). Thus, it is important to be able to predict individuals who might develop LN.

Genetic factors play an important role in the pathogenesis of LN (6). Most studies aiming to identify susceptibility genes for LN are candidate gene association analyses. In such studies, SLE-associated genes, mostly related to immunology/inflammation, are often selected to examine their role in LN. However, this approach might lead to the omission of a large number of genes with other roles in the pathogenesis of LN. Genome-wide association studies (GWAS) can explore susceptibility genes in the whole genome and have been successfully applied to multiple complex diseases.

We aimed to find susceptible candidates of LN across the whole genome in the Han Chinese population. We previously performed a GWAS for SLE by genotyping 1,047 SLE cases and 1,205 healthy controls by using Illumina Human610-Quad BeadChips in a Han Chinese population (7). Based on this GWAS data set we performed whole genome single-nucleotide polymorphism (SNP)- and gene -based analyses for the first time to explore LN susceptibility polymorphisms, genes, or loci in the Han Chinese population in the present study.

## Material and methods

### Subjects and phenotype definition

All the samples used in this study were obtained from multiple hospitals in China. Clinical information was collected from questionnaires and clinical records. Informed consent was obtained from all participants, and the study was approved by the institutional review board at each institution.

In this study, LN patients were cases, while the SLE without LN were the controls, who were genetically comparable to the LN patients. All patients met the American College of Rheumatology criteria (1997) (8). As shown in **Table 1**, there were no significant differences in sex or age between the two groups. LN was diagnosed according to the renal sub-phenotype data using ACR classification (proteinuria of >500 mg/24 hours or >3+, or the presence of cellular casts, or diagnosed as LN by renal biopsy). SLE without LN was diagnosed as SLE without the above renal disorder. There were 592 LN and 453 SLE without LN patients in the discovery stage, 188 LN and 171 SLE without LN patients in the replication study, 6 LN patients and 6 healthy controls in the quantitative real-time PCR experiment and 39 LN patients and 35 healthy controls in the Enzyme-linked immunosorbent assay experiment.

**Table 1:** Characteristics of patients in the study.

Characteristics		Discovery stage		SNP replication	
		LN (n=592)	SLE without LN (n=453)	LN (n=188)	SLE without LN (n=171)
**Female**	557(94.1%)	425(93.8%)	155 (82.4%)	150 (87.7%)
**Age (year)**	35(24-43)	34(24-42)	32 (24-44)	39 (27-48)*

*Compared with the LN group *P<0.05.*

Results are shown as means ± SD or median (inter-quartile range) according to the distribution.

### Genotyping and quality controls in the discovery stage

This analysis was based on genotyping data from the Chinese SLE GWAS, including 1,099 SLE cases. As described previously, SNP genotyping was performed using Illumina Human 610-Quard BeadChips (7).

A total of 1,099 SLE cases were genotyped initially. Cases with genotyping call rates less than 98% or repeated or related samples were removed (52 SLE patients); 2 SLE cases without renal information were also removed. Subsequently, principal component analysis (PCA) was performed to assess genetic heterogeneity using EIGENSTRAT. No outlier was revealed by the PCA plots (**S1 Figure**). In total, 1,045 SLE patients (592 LN patients and 453 SLE without LN patients) were included in genome-wide association analysis.

All SNPs on the X, Y and mitochondrial chromosomes were removed, after which SNPs with unclear cluster patterns of the genotyping data were removed. SNPs with genotyping call rates less than 90% or Hardy-Weinberg equilibrium *P <*10^-7^ were also removed. Thus, 492,970 SNPs were included in genome-wide association analysis.

### Imputation

Genotypes were imputed on the basis of the 1000 Genomes Project phase 3 (hg19) using the IMPUTE program (v2.0). A total of 14,076,911 SNPs were imputed. A quality control was applied for the imputed SNPs: all SNPs on the X, Y and mitochondrial chromosomes were removed. SNPs with genotyping call rates less than 90% or Hardy-Weinberg equilibrium *P <*10^-7^ were also removed.

### SNP selection for replication analysis

As the *P* values of the imputed SNPs were also modest, the SNP selection for the replication study was based on the results of genome-wide association analysis without imputation. SNPs with *P*<5×10^-4^ in the genome-wide association analysis were selected, and those with high *P* values of Hardy-Weinberg equilibrium (*P*>5×10^-4^ in controls), high call rates (>95%), high minor allele frequencies (>0.05 both in cases and controls), and proximity to genes associated with LN pathogenesis (immunology, inflammation, renal resident cell, and so on) remained. The top one or two tag SNPs with the lowest *P* value were selected when multiple SNPs were localized to one distinct genomic locus based on physical location. Ultimately, 56 SNPs in 34 loci were selected for replication analysis.

### Genotyping and quality controls in the replication study

The 56 SNPs were genotyped in 191 LN patients and 171 SLE without LN patients by using the Agena MassARRAY system. Agena MassARRAY Assay Design 3.1 software was used to design a multiplexed SNP MassEXTEND assay, and SNP genotyping was performed using Agena MassARRAY RS1000 with the manufacturer’s protocols. Agena Typer 4.0 software was employed to perform data management and analyses. Three cases with call rates <90% were excluded. Two SNPs were excluded because of a call rate <90% or a *P* value of Hardy-Weinberg equilibrium <0.001. The remaining SNP cluster patterns from the genotyping data were checked to confirm their good quality. After quality control, 54 SNPs in 188 LN patients and 171 SLE without LN patients were remained in the replication stage analysis.

### Association analysis

In the discovery stage, association analyses were performed with PLINK using logistic regression with age and sex as covariates. In the replication study, the 54 SNPs were analysed with the same association test. To combine the association evidence from the GWAS and SNP replication samples, a joint analysis of all combined samples was performed using meta-analysis with PLINK. SNPs with *P*<5×10^−8^ were considered genome-wide significant, and those with *P*<10^−4^ were considered as suggestive association.

### Bioinformatics analysis and expression analysis

To evaluate the potential biological function of the most significant SNP, rs12606116, and related genes, we used QTLbase (http://mulinlab.org/qtlbase/), GTEx (https://gtexportal.org/home/), and GEO (https://www.ncbi.nlm.nih.gov/geoprofiles/) databases to conduct expression quantitative trait locus (eQTL) analysis and gene expression difference analysis. The gene expression data of LN and normal kidney tissues were downloaded from the GEO database. Those genes around rs12606116 ± 400 kb were focused on. The eQTL data of rs12606116 and genes around rs12606116 ± 400 kb were downloaded from QTLbase and GTEx databases.

### Gene-based and gene set enrichment analysis

Gene-based analysis was performed with the versatile gene-based test for GWAS (VEGAS), which uses SNP-level data to incorporate information from a full set of markers annotated to each gene and accounts for linkage disequilibrium (LD) between markers (9, 10). The association *P*-values of a given gene with n SNPs were converted to upper tail chi-square statistics with 1 degree of freedom. Then the empirical gene-based *P*-value was calculated using the Monte Carlo simulation (11).

To explore the biological functions and cellular signaling pathways, all genes with *P*<0.05 in the gene-based test were used as input to perform gene set enrichment analysis using Gene Ontology (GO) and Kyoto Encyclopedia of Genes and Genomes (KEGG). GO analysis was performed in three aspects: biological process (BP), cellular component (CC), and molecular function (MF). GO analysis and KEGG pathway analysis were both conducted by the Database for Annotation, Visualization, and Integrated Discovery (DAVID).

### Animals

Three 6-8 weeks old MRL/lpr mice were purchased from Shanghai Model Organisms and subsequently maintained in the animal facility at the Chinese People’s Liberation Army General Hospital. Mice were monitored for lupus for 12 weeks, at which time they were sacrificed for renal tissue. Renal tissue from three BALB/c mice were served as control. All animal experiments conducted have been approved by the Animal Ethics Committee of the Chinese People’s Liberation Army General Hospital.

### Quantitative real-time PCR

The gene expression differentiation of ADCYAP1 and LINC00470 genes was analysed in peripheral blood mononuclear cell (PBMC) samples of 6 LN patients and 6 healthy controls. Besides, the gene expression of transforming growth factor beta receptor 2 (TGFBR2) gene was analysed in renal tissue of 3 MRL/lpr lupus mice and 3 BALB/c mice. RNA templates were extracted from PBMC or renal tissue samples with TRIzol. cDNA was synthesized from 1 μg of RNA templates using reverse transcriptase and oligo(dT) primers (NEB). The sequences of the primers used for amplification are given in **S1 Table**. 18S was used as a reference for normalization. The qRT-PCR reaction was carried out in triplicate using SYBR Green on an ABI7500 Real-Time PCR System.

### Enzyme-linked immunosorbent assay

Human transforming growth factor beta1 ELISA Kit (ab100647; Abcam) was used to measure plasma levels of transforming growth factor beta1 (TGF-β1) according to the manufacturer’s instructions.

### Statistical analysis

Data were analysed using SPSS 21.0 (SPSS, Chicago, IL, USA) statistical software. Continuous variables were expressed as median (inter-quartile range) or mean ± standard deviation according to the distribution, and categorical variables were expressed as frequency (percentage). Differences between the two groups were examined using the Mann-Whitney U-test or Student’s t-test. For categorical variables, differences between groups were analysed using the χ^2^ test. *P* values<0.05 were considered statistically significant.

## Results

### Genome-wide association of LN

There were 592 LN patients and 453 SLE without LN patients included in the GWAS discovery stage. As shown in **Table 1**, there were no significant differences in sex or age between the two groups.Manhattan and quantile-quantile plots (q-q plots) are shown in **S2 Figure** (492,970 tag SNPs). The genome inflation factor (λ_GC_) of 1.018 indicated adequate control for population stratification. Although no SNP achieved genome-wide significance (*P*< 5×10^−8^), 44 SNPs achieved suggestive significance (*P*< 10^−4^) (**S2 Table**). The most significant SNP was rs10151371 (*P*=9.90×10^−6^) at 14q31.3.

### Imputation

**Figure 1:**
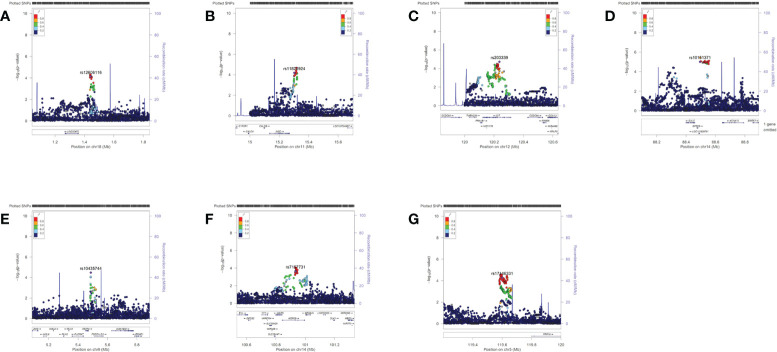
Region plots of the genome-wide association results of the suggestive single-nucleotide polymorphisms (SNPs). These region plots were made in LocusZoom (http://locuszoom.sph.umich.edu) with hg19/1000 Genomes Nov 2014 ASN as the LD Population. The selected SNP was in purple and its linkage disequilibrium values (r2) with nearby SNPs are indicated by different colours. **(A)** rs12606116 ± 400 kb (18p11.32) **(B)** rs11826924 ± 400 kb (11p15.2) **(C)** rs203339 ± 400 kb (rs433091, 12q24.23) **(D)** rs10151371 ± 400 kb (rs17124022, 14q31.3) **(E)** rs10435744 ± 400 kb (9p24.1) **(F)** rs7157731 ± 400 kb (14q32.2) **(G)** rs17146331 ± 400 kb (5q23.1). The Y axis was -log10 P values.

A total of 14,076,911 SNPs were imputed. After quality control, the most significant imputed SNP was rs117609374 (*P*=3.67×10^−6^) at 14q31.3. Considering the modest *P* value of the imputation results, only the imputed region plots of the 9 SNPs from 7 loci described below are presented in **Figure 1**.

### Replication study

To assess the association of possible SNPs with LN, 56 SNPs in GWAS were selected for genotyping in another 191 LN and 171 SLE without LN patients. After quality control, 54 SNPs in 188 LN patients and 171 SLE without LN patients were analysed. Due to limited power of the replication samples, only one SNP rs12606116 exhibits *P*< 0.05 (**S3 Table**).

### Meta-analysis of the GWAS and replication results

In the meta-analysis of the GWAS and SNP replication results, 9 SNPs showed a suggestive association with LN, at *P*< 10^-4^ (**Table 2**). The most significant SNP was rs12606116 (*P*=8.72×10^−6^), located at 18p11.32. This SNP localizes to an intergenic region close to LINC00470, a long intergenic non-protein coding RNA gene, and the ADCYAP1 gene, which encodes a potent renoprotective peptide. The *P* values of the remaining 8 SNPs were between 10^-5^ and 10^-4^ (**Table 2**). Region plots of the 9 SNPs from 7 loci are presented in **Figure 1**.

**Table 2:** Summary results of 9 SNPs in the GWAS, replication and meta-analysis.

						Meta		GWAS						Replication			
SNP	Chr	Locus	Position	Gene	Allele	*P*	OR	MAF in case	MAF in con	*P*	OR	SE	95%	*P*	OR	SE	95%
													CI				CI
**rs12606116**	18	18p11.32	1443317	ADCYAP1/	T/C	8.72E-06	0.67	0.199	0.275	7.43E-05	0.67	0.1	0.54-0.81	4.17E-02	0.7	0.18	0.50-0.99
				LINC00470													
**rs11826924**	11	11p15.2	15301762	INSC	C/T	1.71E-05	0.71	0.35	0.434	6.53E-05	0.69	0.09	0.57-0.83	9.13E-02	0.76	0.16	0.56-1.04
**rs203339**	12	12q24.23	120236754	CIT	T/C	2.37E-05	1.4	0.451	0.357	1.91E-05	1.49	0.09	1.24-1.78	2.66E-01	1.18	0.15	0.88-1.59
**rs10151371**	14	14q31.3	88494575	GPR65	A/G	2.59E-05	1.73	0.139	0.075	9.90E-06	1.94	0.15	1.45-2.61	4.59E-01	1.22	0.26	0.73-2.04
**rs17124022**	14	14q31.3	88531526	GPR65	C/T	3.15E-05	1.6	0.182	0.11	1.28E-05	1.76	0.13	1.37-2.27	4.51E-01	1.19	0.23	0.76-1.85
**rs10435744**	9	9p24.1	5486856	CD274	G/A	3.93E-05	1.37	0.502	0.406	3.32E-05	1.45	0.09	1.21-1.72	3.05E-01	1.17	0.15	0.87-1.58
**rs433091**	12	12q24.23	120228792	CIT	C/T	4.03E-05	1.37	0.49	0.402	9.88E-05	1.42	0.09	1.19-1.69	1.43E-01	1.25	0.15	0.93-1.69
**rs7157731**	14	14q32.2	100935129	WDR25	G/A	7.77E-05	0.73	0.327	0.405	2.29E-04	0.71	0.09	0.59-0.85	1.29E-01	0.78	0.16	0.57-1.07
**rs17146331**	5	5q23.1	119600027	PRR16	T/G	8.95E-05	0.73	0.378	0.465	5.16E-05	0.69	0.09	0.57-0.82	3.70E-01	0.87	0.15	0.65-1.17

Allele: Minor/Major allele; GWAS: genome wide association study; MAF in case: minor allele frequency in 592 LN patients; MAF in con: minor allele frequency in 453 SLE without LN patients

### qRT-PCR

**Figure 2:**
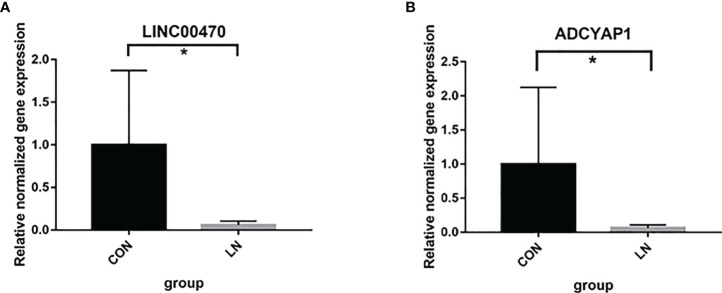
mRNA expression of LINC00470 **(A)** and ADCYAP1 **(B)** in PBMCs of 6 LN patients and 6 healthy controls assessed by qRT-PCR. *P<0.05.

Further studies were applied to verify the biological function of rs12606116 and related genes. Data in the GEO database showed that mRNA expression of ADCYAP1, which was the closest coding gene to rs12606116, was significantly different in the renal tubules of LN patients and healthy controls (GSE32591, *P*=1.06×10^−3^, logFC=0.16). The QTLbase database showed an association between rs12606116 and LINC00470, which was the closest gene, in CD4+ T cells and CD14+ monocytes cells. Thus, we measured the mRNA levels of LINC00470 and ADCYAP1 in PBMCs of 6 LN patients and 6 healthy controls using qRT-PCR. As shown in **Figure 2**, the expression of LINC00470 and ADCYAP1 in the PBMCs of LN patients was significantly lower than that in healthy controls. These results further indicated LINC00470 and ADCYAP1 in 18p11.32 might be involved in the development of LN.

### Results of gene-based and gene set enrichment analysis

To explore additional susceptibility genes associated with LN, we optimally use the GWAS data sets to conducted gene-based analysis using VEGAS. In the gene-based analysis, the SNPs in the GWAS were mapped to 14,012 genes (nSNP>1). In total, 690 genes showed evidence of association with LN (*P*<0.05), including LINC00470, identified by the above SNP-based analysis (**S4 Table)**.

**Table 3** shows the top 10 genes ranked by their *P*-values from the VEGAS analysis. The topmost hit was LINC01146 with *P*=1.20×10^−5^. Two of the 10 genes also featured in the list from the SNP-based analysis: CIT (*P*=2.78×10^−4^) and WDR25 (*P*=3.39×10^−4^).

**Table 3:** The top 10 genes from the gene-based analysis.

Chr	Gene	nSNPs	Start	Stop	Pvalue	TopSNP	TopSNP.pvalue
14	LINC01146	15	88490893	88553688	1.20E-05	rs10151371	3.68E-06
3	TMEM207	6	190146443	190167665	3.00E-05	rs2378570	2.93E-05
3	FETUB	3	186358148	186370797	1.21E-04	rs4686434	1.43E-04
3	CLDN1	5	190023489	190040235	1.76E-04	rs10513846	2.06E-04
5	EFNA5	78	106712589	107006596	1.79E-04	rs164699	9.04E-04
9	ZBTB26	2	125680377	125693779	2.12E-04	rs3824535	3.16E-04
12	CIT	19	120123594	120315095	2.78E-04	rs203339	1.34E-05
14	WDR25	9	100842754	100996640	3.39E-04	rs7157731	2.24E-04
9	RABGAP1	8	125703287	125867147	4.47E-04	rs10818781	3.00E-04
1	GNL2	5	38032412	38061586	4.49E-04	rs7535432	1.37E-04

**Figure 3:**
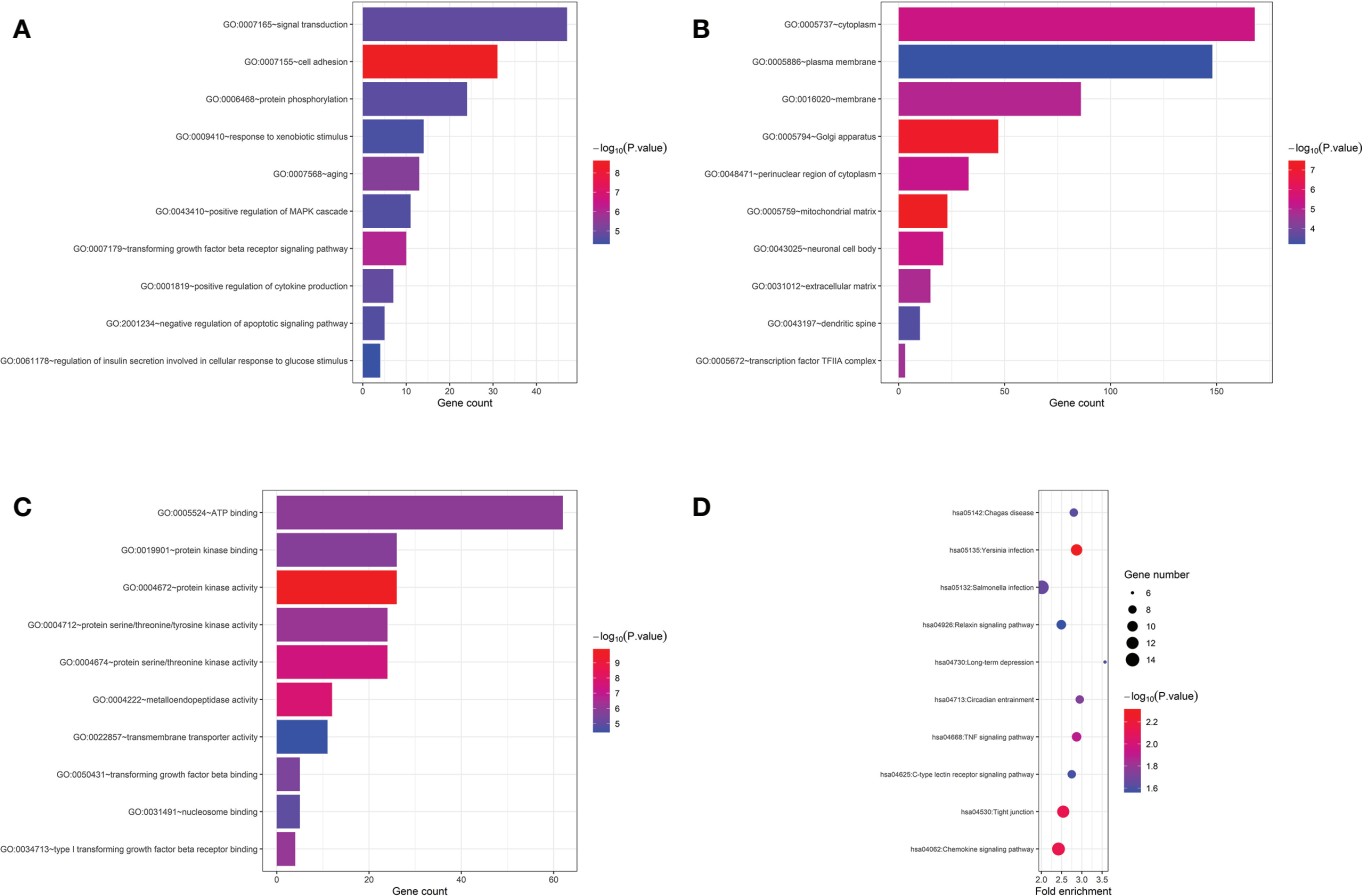
GO and pathway enrichment analysis of genes with P<0.05 in gene-based analysis. **(A)** top 10 biological process GO terms. **(B)** top 10 cellular component GO terms. **(C)** top 10 molecular function GO terms. **(D)** top 10 KEGG pathway terms.

Furthermore, we carried out enrichment analysis with all the 690 genes those presented *P<*0.05 in the gene-based analysis. The ten most significant GO terms and pathways are shown in **Figure 3**. Top associated GO terms included “cell adhesion”, “positive regulation of MAPK cascade”, “protein kinase activity”, “Chemokine signaling pathway”, “TNF signaling pathway”, and so on. Interestingly, “transforming growth factor beta receptor signaling pathway”, ‘‘type I transforming growth factor beta receptor binding’’ and ‘‘transforming growth factor beta binding’’ were significantly enriched, highlighting the involvement of transforming growth factor beta (TGF-β) signalings in the pathogenic mechanisms of LN.

### Detection of related molecules in TGF-β signaling pathway

**Figure 4:**
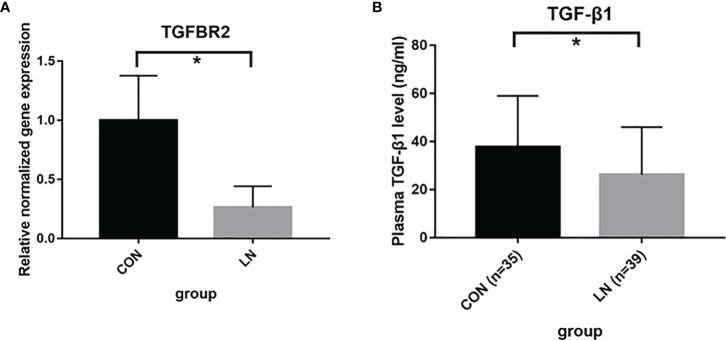
Detection of TGFBR2 and TGF-β1 in TGF-β signaling pathway. **(A)** mRNA expression of TGFBR2 in renal tissue of 3 MRL/lpr mice (LN) and 3 BALB/c mice (control) assessed by qRT-PCR. **(B)** the plasma levels of TGF-β1 in 39 LN patients and 35 healthy controls assessed by ELISA. *P<0.05.

We further examined the key molecules, TGFBR2 and TGF-β1, in TGF-β signaling pathway. The MRL/lpr mouse is a spontaneous disease model for complement-associated inflammatory kidney disease, similar to LN (12, 13). Thus, qRT-PCR was performed to assess the expression of TGFBR2 gene in renal tissue of 3 MRL/lpr mice (LN group) and 3 BALB/c mice (control group). As shown in **Figure 4A**, the expression of TGFBR2 gene in the LN group was significantly lower than that in controls. Then the plasma levels of TGF-β1 were detected using ELISA in 39 LN patients and 34 healthy controls. It was observed that plasma levels of TGF-β1 was significantly lower in patients with LN comparing to control group (**Figure 4B**).

## Discussion

We performed GWAS of SNP- and gene- based analyses to identify susceptibility SNPs, genes and loci associated with LN in the Han Chinese population for the first time. In the SNP-based GWAS results, 9 SNPs showed suggestive associations with LN (*P*< 10^−4^), among which rs12606116 was the most significant SNP. Subsequently, bioinformatics analysis and qRT-PCR research verified the mRNA levels of LINC00470 and ADCYAP1, the closest genes to rs12606116, in PBMCs were significantly lower in LN patients than in healthy controls. Gene-based analysis further validates the association of LINC00470 with LN. These results indicated that LINC00470 and ADCYAP1 in 18p11.32 might be involved in the development of LN. Besides LINC00470, the gene-based analysis found 690 genes associated with LN (*P*<0.05). The subsequent gene set enrichment analysis identified several pathways involved in LN, especially the signalings of TGF-β. Lower plasma level of TGF-β1 in LN patients and lower expression of TGFBR2 in lupus mice kidney futher indicate the involvement of TGF-β in LN. This first GWAS of LN in the Han Chinese population provide several promising susceptibility candidates involved in LN.

Two GWASs were performed to identify loci predisposing individuals towards LN. Chung et al. completed a meta-analysis of three GWASs of SLE, containing 588 European women with LN and 1,412 SLE women without LN, to identify LN-associated loci (14). The strongest evidence for association was observed localizing to 4q11-q13 (PDGFRA, *P*=4.5×10^−7^). Chen et al. performed a meta-analysis of two GWASs of European SLE (1,152 LN and 1,949 SLE without LN patients), they also did not identify any genome-wide loci significantly associated with LN (*P*< 5×10^−8^) (15). Similar to the two GWASs, we did not find SNPs with *P*< 5×10^−8^. The less significance of those results might be due to the control group, namely, SLE patients without renal damage. Some of these patients in the control group might already have kidney damage without clinical manifestations, or they might develop LN in the future. All these factors potentially reduce statistical power and increase type 2 error rather than false positive findings. Nonetheless, there is a clear trend in our results. We identified nine new suggestive SNPs associated with LN (*P*< 10^−4^). The most significant SNP was rs12606116 (*P*=8.72×10^-6^), which is located close to LINC00470 and ADCYAP1 at 18p11.32, the new locus reported in LN GWASs. Thus, we believe that the results is a new suggestive locus for LN in the Han Chinese population.

The qRT-PCR replication research in the PBMCs further illustrated our findings. In the biological analysis, we found that the mRNA expression of ADCYAP1 was different in the renal tubules of LN patients and healthy controls. The QTLbase database also showed an association between rs12606116 and LINC00470. Further qRT-PCR replicated that the expression of LINC00470 and ADCYAP1 in the PBMCs of LN patients was significantly lower than that of healthy controls. Especially, LINC00470 was also identified in the gene-based analysis.

LINC00470 is a long intergenic non-protein coding RNA gene and is affiliated with the lncRNA class. Noncoding RNAs, particularly microRNAs and lncRNAs, have been implicated in multiple biological processes in normal and various diseases. There are few reports about this gene. As reported, LINC00470 is a new AKT activator that mediates glioblastoma cell autophagy (16). It also facilitates malignant proliferation in hepatocellular carcinoma, lung cancer, and gastric cancer (17–19). However, its function in LN needs further study.

ADCYAP1 encodes pituitary adenylate cyclase-activating polypeptide (PACAP), which is a multifunctional neuropeptide that might act as a potent renoprotective peptide (20). Experiments in PACAP-deficient mice showed that a lack of endogenous PACAP leads to higher susceptibility to *in vivo* renal ischaemia/reperfusion, suggesting that PACAP protects the kidney against oxidative stress (21, 22). In addition, PACAP may have an immunomodulatory role. As reported, PACAP was widely distributed in the organism including the immune system, such as thymus, lymph nodes and spleen. While exposed to proinflammatory stimuli, PACAP may prevent inflammation by down-regulating a series of cytokines and chemokines produced by innate immune cells (such as macrophages). It has been also shown that PACAP can participate in the immune response by inducing the production of regulatory T cells (23, 24).

Interestingly, two genes (CIT and WDR25) showed suggestive association with LN in both the SNP-based and gene-based analyses. CIT gene encodes a serine/threonine-protein kinase that functions in cell division. CIT has been reported to play a role in cancer proliferation and central nervous system development (25). WDR25 encodes a protein containing 7 WD repeats, involved in cell cycle progression, signal transduction, apoptosis, and gene regulation (26). Gene-based analysis also highlighted several other genes, including a gene implicated in polycystic kidney disease (TMEM207), a gene expressed at the tight junction of renal tubule epithelial cells (CLDN1), and a gene associated with cell proliferation (GNL2) (27–29). It would be interesting to see whether these genes could be replicated in future studies.

Our enrichment analysis revealed several pathways, especially the TGF-β signaling pathways, including “transforming growth factor beta receptor signaling pathway”, ‘‘type I transforming growth factor beta receptor binding’’ and ‘‘transforming growth factor beta binding’’. There have been many studies, including cohorts and animal models, suggesting the involvement of TGF-β, especially TGF-β1, in SLE. Our results also highlighted the important role of TGF-β in LN. TGF-β signalings regulate various biological responses, including proliferation, migration, differentiation, apoptosis, and the immune response (30). There are three TGF-β forms (TGF-β1, 2, and 3) and three TGF-β receptors (TGFBR1, 2, and 3). Among them, TGF-β1 was the first fully cloned of the TGF-β superfamily members, and was crucial in the signaling pathway (31). Besides, TGFBR2 is the receptor directly bounds to TGF-β, and thus it is also a key molecule for activation of downstream signaling (30). To verify the involvement of TGF-β in LN, we further detected TGFBR2 and TGF-β1. We found that TGFBR2 mRNA expression decreased in the LN mice kidneys, and plasma TGF-β1 levels also decreased in LN patients. As reported, SLE/LN patients often produce lower levels of TGF-β1 when compared with healthy individuals (32–36). However, there are limited studies on the mechanism of TGFBR2 in LN. Saxena et al. reported that expression of TGF-β receptor type II was increased in the kidneys of autoimmune-prone BWF1 mice (32). Differences in animal models might partly explain the opposite results. Taken together, these findings in our study indicate that reduced TGF-β production might predispose for the development of lupus and target organ damage. However, those association need to be verified in larger sample and the mechanisms remains to be further tested.

Over the past decade, an explosion of SLE GWASs in different races have greatly improved our understanding of the genetic basis of SLE. Over 100 susceptibility loci and hundreds of SNPs for polygenic, multifactorial SLE have been identified. We systematically collected 405 SLE associated SNPs from the GWAS catalogue (https://www.ebi.ac.uk/gwas/) and previous reported SLE GWASs. Among them, 144 SNPs were genotyped in this study. Results of the 144 known SLE susceptible SNPs in this LN GWAS were listed in **S5 Table**. Unexpectedly, only 9 SNPs showed *P*<0.05 in this LN GWAS, and only 3 SNPs had consistant OR (rs4622329, rs3093030, rs11117432). Furthermore, genes showed in **Table 2** and **Table 3** also have not been identified in SLE GWASs. It might suggest that, SNPs those play important roles in the pathogenesis of SLE might not play an equally critical role in LN. There might be some differences between SNPs involved in SLE vs. in LN. We further paid attention to the monogenic causes of SLE and the pathways involved. As reviewed by Demirkaya, there have been more than 30 genes causing the monogenic form of SLE identified, involved in pathways of complement, Type I IFN, self-tolerance, RAS, and so on (37). One patient with NS/Noonan-related syndrome phenotype and SLE characteristics had been reported to have a PTPN11 mutation (37). Coincidentally, PTPN11 gene showed a *P* value<0.05 in our gene-based analysis. As a member of the RAS/MAPK cascade, PTPN11 plays pivotal roles in cell proliferation, differentiation, survival and cell death (38). As we know, MAPK pathway is closely related to renal physiology and pathophysiology (39). The “positive regulation of MAPK cascade” was also enriched in our analysis (**Figure 3**). We speculate that PTPN11 might be involved in LN by activating MAPK downstream cytokine and pathways. The mechanism remains to be verified. Mutation in TNFAIP3, encoding the NF-kB regulatory protein A20, was also reported to associated with SLE (40). TNFAIP3 is an ubiquitin-editing enzyme with a critical function in the inhibition of key proinflammatory molecules, mainly functions as an endogenous regulator of inflammation through termination of nuclear factor (NF)-κB activation (41). TNFAIP3 is downstream of TNF signaling pathway and can be induced by TNF. In our enrichment analysis, TNF signaling pathway was also enriched, indicating that TNFAIP3 and other members of TNF signaling pathway might play a role in the pathogenesis of lupus and LN (**Figure 3**).

There are some limitations in this study. First, the sample size in the discovery stage and SNP replication study was not appreciable enough. With 1,404 SLE participants, we had limited power, at a genome-wide significance level, to uncover an association with small-to-moderate effect sizes. Studies with larger sample sizes or multi-team cooperation of GWAS meta-analyses might be helpful for identifying significant loci. Second, as mentioned above, the control group, namely, SLE patients without renal damage, was clinically evaluated, and some of these patients might already have kidney damage without clinical manifestations. In addition, some patients might develop LN in the future. All these factors potentially reduce statistical power and increase type 2 error rather than false positive findings; thus, they do not decrease the robustness of the associations reported here. Thirdly, compared with SLE without LN patients, the heritability of LN might not be significant enough. Some variants with lower allele frequencies and weaker genetic effects that are difficult to identify might exist. Finally, the expression data of ADCYAP1 and LINC00470 genes in the kidney was not available. We have tried to analyse the gene expression differentiation of the two genes in kidney tissues of lupus prone mice. However, the expression of the two genes in kidney was too low to acquire in the qRT-PCR. Thus, we collected the PBMC, which included both the CD4+ T cells and CD14+ monocytes cells. Moreover, larger sample sizes in the qRT-PCR and ELISA parts might be needed to verify the differences.

## Conclusion

In summary, we performed SNP- and gene-based GWAS of LN for the first time in the Han Chinese population and found some promising candidates that exhibit suggestive associations with LN. In particular, the association between rs12606116 in 18p11.32 and LN was replicated, indicating that it was a new suggestive locus for LN in the Han Chinese population. We also identified several other promising genes and pathways for LN, especially the involvement of TGF-β pathways. These findings enrich our understanding of LN inheritance in the Han Chinese population and provide important clues for future genetic research on LN. However, further exploration in the future is warranted.

## Data availability statement

According to national legislation/guidelines, specifically the Administrative Regulations of the People’s Republic of China on Human Genetic Resources (http://www.gov.cn/zhengce/content/2019-06/10/content_5398829.htm, http://english.www.gov.cn/policies/latest_releases/2019/06/10/content_281476708945462.htm), no additional raw data is available at this time. Data of this project can be accessed after an approval application to the China National Genebank (CNGB, https://db.cngb.org/cnsa/). Please refer to https://db.cngb.org/search/project/CNP0003504/, or email: CNGBdb@cngb.org for detailed application guidance. The accession id is CVF0109356.

## Ethics statement 

The studies involving human participants were reviewed and approved by the PLA institutional review board. Written informed consent to participate in this study was provided by the participants’ legal guardian/next of kin.

## Author contributions

KS and XDZ contributed equally to this work. XC, XJZ, XS, GC and QL conceived the study design. KS, XDZ, YS, LL and LW wrote the manuscript. KS and XDZ conducted all of the analyses. XL, SS, YD, QO, PC, GC, MC, YZ, BL and JZ managed the cohort data. All authors contributed to the article and approved the submitted version.

## Funding

This work was supported by the National Natural Science Foundation of China [grant number 81830019]; the Haihe Laboratory of Cell Ecosystem Innovation Fund [grant number HH22KYZX0003]; the National Natural Science Foundation of China [81872527, 81803117]; and Beijing Municipal Natural Science Foundation [grant number 7202188].

## Conflict of interest

The authors declare that the research was conducted in the absence of any commercial or financial relationships that could be construed as a potential conflict of interest.

## Publisher’s note

All claims expressed in this article are solely those of the authors and do not necessarily represent those of their affiliated organizations, or those of the publisher, the editors and the reviewers. Any product that may be evaluated in this article, or claim that may be made by its manufacturer, is not guaranteed or endorsed by the publisher.

## References

[1] Durcan L O'Dwyer T Petri M. Management strategies and future directions for systemic lupus erythematosus in adults. Lancet (2019) 393(10188):2332–43. doi: 10.1016/S0140-6736(19)30237-5 31180030

[2] Jha SB Rivera AP Flores Monar GV Islam H Puttagunta SM Islam R. Systemic lupus erythematosus and cardiovascular disease. Cureus (2022) 14(2):e22027. doi: 10.7759/cureus.22027 35282557 PMC8910778

[3] Maroz N Segal MS. Lupus nephritis and end-stage kidney disease. Am J Med Sci (2013) 346(4):319–23. doi: 10.1097/MAJ.0b013e31827f4ee3 23370533

[4] Almaani S Meara A Rovin BH. Update on lupus nephritis. Clin J Am Soc Nephrol (2017) 12(5):825–35. doi: 10.2215/CJN.05780616 PMC5477208 27821390

[5] Yap DY Tang CS Ma MK Lam MF Chan TM. Survival analysis and causes of mortality in patients with lupus nephritis. Nephrol Dial Transplant (2012) 27(8):3248–54. doi: 10.1093/ndt/gfs073 22523116

[6] Iwamoto T Niewold TB. Genetics of human lupus nephritis. Clin Immunol (2017) 185:32–9. doi: 10.1016/j.clim.2016.09.012 PMC5373939 27693588

[7] Han JW Zheng HF Cui Y Sun LD Ye DQ Hu Z. Genome-wide association study in a Chinese han population identifies nine new susceptibility loci for systemic lupus erythematosus. Nat Genet (2009) 41(11):1234–7. doi: 10.1038/ng.472 19838193

[8] Hochberg MC. Updating the American college of rheumatology revised criteria for the classification of systemic lupus erythematosus. Arthritis Rheum (1997) 40(9):1725. doi: 10.1002/art.1780400928 9324032

[9] Neale BM Sham PC. The future of association studies: Gene-based analysis and replication. Am J Hum Genet (2004) 75(3):353–62. doi: 10.1086/423901 PMC1182015 15272419

[10] Liu JZ McRae AF Nyholt DR Medland SE Wray NR Brown KM. A versatile gene-based test for genome-wide association studies. Am J Hum Genet (2010) 87(1):139–45. doi: 10.1016/j.ajhg.2010.06.009 PMC2896770 20598278

[11] Gao J Zhu C Zhang Y Sheng Y Yang F Wang W. Association study and fine-mapping major histocompatibility complex analysis of pemphigus vulgaris in a han Chinese population. J Invest Dermatol (2018) 138(11):2307–14. doi: 10.1016/j.jid.2018.05.011 29857070

[12] Wenderfer SE Soimo K Wetsel RA Braun MC. Analysis of C4 and the C4 binding protein in the Mrl/Lpr mouse. Arthritis Res Ther (2007) 9(5):R114. doi: 10.1186/ar2320 17971229 PMC2212569

[13] Watson ML Rao JK Gilkeson GS Ruiz P Eicher EM Pisetsky DS. Genetic analysis of mrl-lpr mice: Relationship of the fas apoptosis gene to disease manifestations and renal disease-modifying loci. J Exp Med (1992) 176(6):1645–56. doi: 10.1084/jem.176.6.1645 PMC2119463 1460423

[14] Chung SA Brown EE Williams AH Ramos PS Berthier CC Bhangale T. Lupus nephritis susceptibility loci in women with systemic lupus erythematosus. J Am Soc Nephrol (2014) 25(12):2859–70. doi: 10.1681/ASN.2013050446 PMC4243339 24925725

[15] Chen L Wang YF Liu L Bielowka A Ahmed R Zhang H. Genome-wide assessment of genetic risk for systemic lupus erythematosus and disease severity. Hum Mol Genet (2020) 29(10):1745–56. doi: 10.1093/hmg/ddaa030 PMC7322569 32077931

[16] Liu C Zhang Y She X Fan L Li P Feng J. A cytoplasmic long noncoding rna Linc00470 as a new akt activator to mediate glioblastoma cell autophagy. J Hematol Oncol (2018) 11(1):77. doi: 10.1186/s13045-018-0619-z 29866190 PMC5987392

[17] Huang W Liu J Yan J Huang Z Zhang X Mao Y. Lncrna Linc00470 promotes proliferation through association with Nf45/Nf90 complex in hepatocellular carcinoma. Hum Cell (2020) 33(1):131–9. doi: 10.1007/s13577-019-00288-8 31612313

[18] Cao Q Dong Z Liu S An G Yan B Lei L. Construction of a metastasis-associated cerna network reveals a prognostic signature in lung cancer. Cancer Cell Int (2020) 20:208. doi: 10.1186/s12935-020-01295-8 32518519 PMC7271455

[19] Yan J Huang X Zhang X Chen Z Ye C Xiang W. Lncrna Linc00470 promotes the degradation of pten mrna to facilitate malignant behavior in gastric cancer cells. Biochem Biophys Res Commun (2020) 521(4):887–93. doi: 10.1016/j.bbrc.2019.11.016 31711642

[20] Brubel R Horvath G Reglodi D Lubics A Tamas A Kiss P. Presence of pituitary adenylate cyclase activating polypeptide and its type I receptor in the rat kidney. Transplant Proc (2011) 43(4):1297–9. doi: 10.1016/j.transproceed.2011.03.081 21620115

[21] Laszlo E Varga A Kovacs K Jancso G Kiss P Tamas A. Ischemia/Reperfusion-induced kidney injury in heterozygous pacap-deficient mice. Transplant Proc (2015) 47(7):2210–5. doi: 10.1016/j.transproceed.2015.07.027 26361682

[22] Szakaly P Laszlo E Kovacs K Racz B Horvath G Ferencz A. Mice deficient in pituitary adenylate cyclase activating polypeptide (Pacap) show increased susceptibility to in vivo renal Ischemia/Reperfusion injury. Neuropeptides (2011) 45(2):113–21. doi: 10.1016/j.npep.2010.12.003 21211837

[23] Bellinger DL Lorton D Horn L Brouxhon S Felten SY Felten DL. Vasoactive intestinal polypeptide (Vip) innervation of rat spleen, thymus, and lymph nodes. Peptides (1997) 18(8):1139–49. doi: 10.1016/s0196-9781(97)00075-2 9396054

[24] Abad C Tan YV. Immunomodulatory roles of pacap and vip: Lessons from knockout mice. J Mol Neurosci (2018) 66(1):102–13. doi: 10.1007/s12031-018-1150-y 30105629

[25] Sahin I Kawano Y Sklavenitis-Pistofidis R Moschetta M Mishima Y Manier S. Citron rho-interacting kinase silencing causes cytokinesis failure and reduces tumor growth in multiple myeloma. Blood Adv (2019) 3(7):995–1002. doi: 10.1182/bloodadvances.2018028456 30940634 PMC6457230

[26] Jin F Dai J Ji C Gu S Wu M Qian J. A novel human gene (Wdr25) encoding a 7-Wd40-Containing protein maps on 14q32. Biochem Genet (2004) 42(11-12):419–27. doi: 10.1023/b:bigi.0000043954.64202.61 15587985

[27] Kito Y Saigo C Takeuchi T. Novel transgenic mouse model of polycystic kidney disease. Am J Pathol (2017) 187(9):1916–22. doi: 10.1016/j.ajpath.2017.05.002 28666097

[28] Angelow S Ahlstrom R Yu AS. Biology of claudins. Am J Physiol Renal Physiol (2008) 295(4):F867–76. doi: 10.1152/ajprenal.90264.2008 PMC2576152 18480174

[29] Iuchi S Paulo JA. Rnametasome network for macromolecule biogenesis in human cells. Commun Biol (2021) 4(1):1399. doi: 10.1038/s42003-021-02928-y 34912035 PMC8674265

[30] Vander Ark A Cao J Li X. Tgf-beta receptors: In and beyond tgf-beta signaling. Cell Signal (2018) 52:112–20. doi: 10.1016/j.cellsig.2018.09.002 30184463

[31] Lodyga M Hinz B. Tgf-Beta1 - a truly transforming growth factor in fibrosis and immunity. Semin Cell Dev Biol (2020) 101:123–39. doi: 10.1016/j.semcdb.2019.12.010 31879265

[32] Saxena V Lienesch DW Zhou M Bommireddy R Azhar M Doetschman T. Dual roles of immunoregulatory cytokine tgf-beta in the pathogenesis of autoimmunity-mediated organ damage. J Immunol (2008) 180(3):1903–12. doi: 10.4049/jimmunol.180.3.1903 PMC2291535 18209088

[33] Sanjabi S Oh SA Li MO. Regulation of the immune response by tgf-beta: From conception to autoimmunity and infection. Cold Spring Harb Perspect Biol (2017) 9(6):a022236. doi: 10.1101/cshperspect.a022236 28108486 PMC5453394

[34] Lee YH Bae SC. Association between circulating transforming growth factor-Beta1 level and polymorphisms in systemic lupus erythematosus and rheumatoid arthritis: A meta-analysis. Cell Mol Biol (Noisy-le-grand) (2017) 63(1):53–9. doi: 10.14715/cmb/2017.63.1.11 28234629

[35] Xiang N Fang X Sun XG Zhou YB Ma Y Zhu C. Expression profile of Pu.1 in Cd4(+)T cells from patients with systemic lupus erythematosus. Clin Exp Med (2021) 21(4):621–32. doi: 10.1007/s10238-021-00717-9 33966135

[36] Rashad NM El-Shabrawy RM Said D El-Shabrawy SM Emad G. Serum levels of transforming growth factor beta -1 (Tgf-Beta1) as an early no invasive marker for diagnosis of lupus nephritis in systemic lupus erythematosus patients. Egypt J Immunol (2019) 26(1):31–42. 31332994

[37] Demirkaya E Sahin S Romano M Zhou Q Aksentijevich I. New horizons in the genetic etiology of systemic lupus erythematosus and lupus-like disease: Monogenic lupus and beyond. J Clin Med (2020) 9(3):712. doi: 10.3390/jcm9030712 PMC7141186 32151092

[38] Xu HY Yuan YY Dai P. Ptpn11 and the deafness. Lin Chung Er Bi Yan Hou Tou Jing Wai Ke Za Zhi (2019) 33(9):830–4. doi: 10.13201/j.issn.1001-1781.2019.09.008 31446698

[39] Tian W Zhang Z Cohen DM. Mapk signaling and the kidney. Am J Physiol Renal Physiol (2000) 279(4):F593–604. doi: 10.1152/ajprenal.2000.279.4.F593 10997909

[40] Aeschlimann FA Batu ED Canna SW Go E Gul A Hoffmann P. A20 haploinsufficiency (Ha20): Clinical phenotypes and disease course of patients with a newly recognised nf-Kb-Mediated autoinflammatory disease. Ann Rheum Dis (2018) 77(5):728–35. doi: 10.1136/annrheumdis-2017-212403 29317407

[41] Mooney EC Sahingur SE. The ubiquitin system and A20: Implications in health and disease. J Dent Res (2021) 100(1):10–20. doi: 10.1177/0022034520949486 32853526 PMC7755949

