# Supplementary material for: Genome-wide association study of SNP- and gene-based approaches to identify susceptibility candidates for lupus nephritis in the Han Chinese population
Source: Front Immunol. 2022 Oct 3;13:908851. doi: 10.3389/fimmu.2022.908851 (PMC9580327; doi:10.3389/fimmu.2022.908851)
Supplement: Supplementary file 1 [file DataSheet_1.pdf]

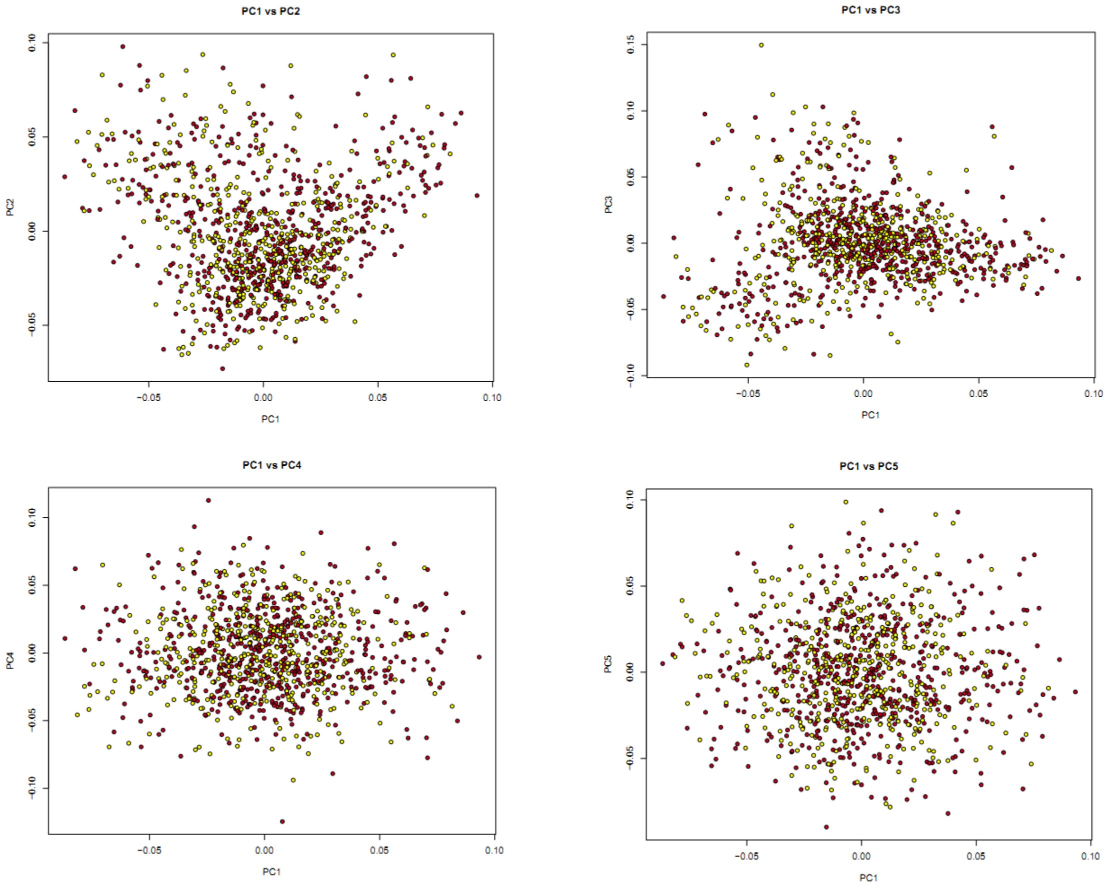

S1 Fig. Principal component analysis plots of the subjects in the genome-wide association study discovery stage. 592 lupus nephritis patients and 453 systemic lupus erythaematosus patients without renal damage are plotted based on eigenvectors 1 to 5 obtained from the PCA analysis using EIGENSTRAT.

A

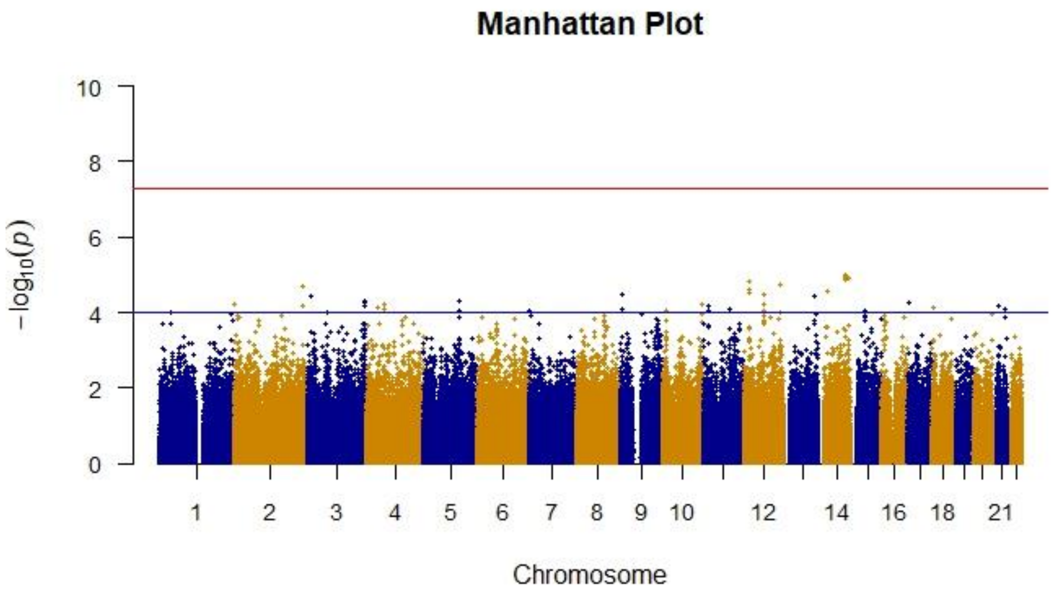

B

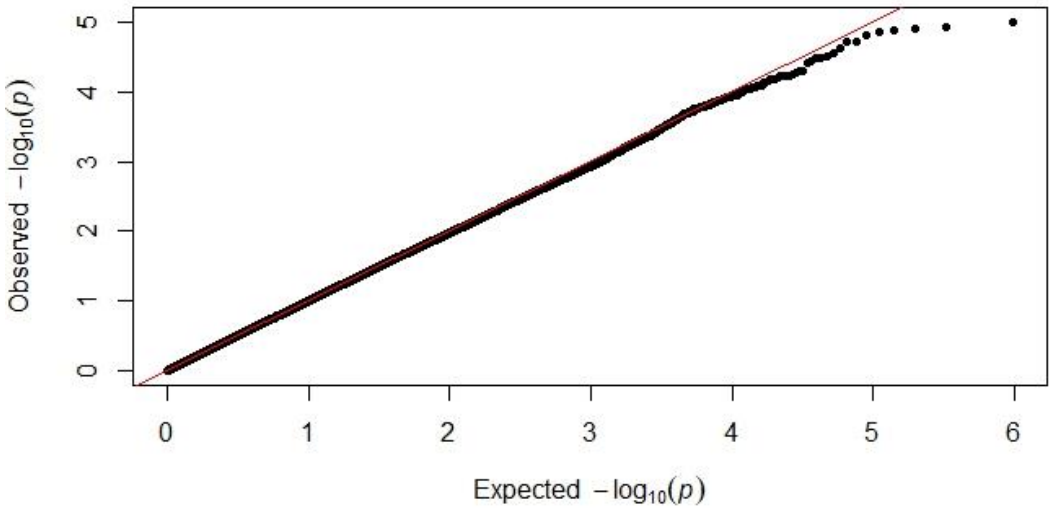

S2 Fig. Manhattan and q-q plot of genome-wide association analysis of lupus nephritis. A. Manhattan plot is a graphical representation of the -log10 *P* values organized by chromosome. The horizontal red line represents $P = 5\times10^{-8}$. The horizontal blue line represents $P = 10^{-4}$. B. The q-q plot compared the -log10 observed *P* values versus the expected -log10 *P* values from association.
